# Inconsistencies between alcohol screening results based on AUDIT-C scores and reported drinking on the AUDIT-C questions: prevalence in two US national samples

**DOI:** 10.1186/1940-0640-9-2

**Published:** 2014-01-27

**Authors:** Kate E Delaney, Amy K Lee, Gwen T Lapham, Anna D Rubinsky, Laura J Chavez, Katharine A Bradley

**Affiliations:** 1 VA HSR&D Denver-Seattle Center of Innovation for Veteran-Centered and Value-Driven Care, Seattle WA, USA; 2 Department of Health Services, University of Washington, Seattle WA, USA; 3 Department of Medicine, University of Washington, Seattle WA, USA; 4 Internal Medicine Residency Program, Brown University, Providence RI, USA; 5 Center of Excellence in Substance Abuse Treatment and Education (CESATE) Veterans Affairs (VA) Puget Sound Health Care System, Seattle WA, USA; 6 Group Health Research Institute, Seattle WA, USA; 7 HSR&D Center for Innovation to Implementation (Ci2i), VA Palo Alto Health Care System, Menlo Park CA, USA

**Keywords:** AUDIT-C, Brief intervention, Unhealthy alcohol use, Alcohol screening, Heavy episodic drinking

## Abstract

**Background:**

The AUDIT-C is an extensively validated screen for unhealthy alcohol use (i.e. drinking above recommended limits or alcohol use disorder), which consists of three questions about alcohol consumption. AUDIT-C scores ≥4 points for men and ≥3 for women are considered positive screens based on US validation studies that compared the AUDIT-C to “gold standard” measures of unhealthy alcohol use from independent, detailed interviews. However, results of screening—*positive or negative based on AUDIT-C scores*—can be inconsistent with *reported drinking* on the AUDIT-C questions. For example, individuals can screen positive based on the AUDIT-C *score* while reporting drinking below US recommended limits on the same AUDIT-C. Alternatively, they can screen negative based on the AUDIT-C *score* while reporting drinking above US recommended limits. Such inconsistencies could complicate interpretation of screening results, but it is unclear how often they occur in practice.

**Methods:**

This study used AUDIT-C data from respondents who reported past-year drinking on one of two national US surveys: a general population survey (N = 26,610) and a Veterans Health Administration (VA) outpatient survey (N = 467,416). Gender-stratified analyses estimated the prevalence of AUDIT-C screen results—*positive or negative screens based on the AUDIT-C score*—that were inconsistent with *reported drinking* (above or below US recommended limits) on the same AUDIT-C.

**Results:**

Among men who reported drinking, 13.8% and 21.1% of US general population and VA samples, respectively, had screening results based on AUDIT-C scores (positive or negative) that were inconsistent with reported drinking on the AUDIT-C questions (above or below US recommended limits). Among women who reported drinking, 18.3% and 20.7% of US general population and VA samples, respectively, had screening results that were inconsistent with reported drinking.

**Limitations:**

This study did not include an independent interview gold standard for unhealthy alcohol use and therefore cannot address how often observed inconsistencies represent false positive or negative screens.

**Conclusions:**

Up to 21% of people who drink alcohol had alcohol screening results based on the AUDIT-C score that were inconsistent with reported drinking on the same AUDIT-C. This needs to be addressed when training clinicians to use the AUDIT-C.

## Background

Alcohol screening and brief interventions (BIs) are effective in reducing primary care patients’ alcohol consumption [1-4]. BIs have been proven effective for patients with unhealthy alcohol use, defined as drinking at levels known to adversely impact health (“risky drinking”) and/or meeting diagnostic criteria for alcohol use disorders (AUD) (Table 1) [5]. The US Preventive Services Task Force (USPSTF) recommends routine implementation of alcohol screening and BIs in primary care to reduce unhealthy alcohol use [3,4].

**Table 1:** **Definitions used in this report and US recommended drinking limits**[39]

		
***Study definition of drinking above recommended limits:***	≥5 drinks^*^ in any single day^**^ and/or exceeding gender-specific US recommendations for maximum average number of drinks^*^ per week	
***Heavy episodic drinking:***	Drinking above recommended daily drinking limits (≥5 drinks in any single day, in this report; gender-specific US recommended limits below)	
***Alcohol use disorder:***	DSM-IV diagnosis of alcohol abuse or dependence	
***Unhealthy alcohol use:***	Drinking above recommended drinking limits and/or alcohol use disorder diagnosis	
***Gender-specific US recommended drinking limits:***	Maximum number of drinks^* ^ ***in any single day***	Maximum average number of drinks^* ^ ***per week***
Men	4 drinks	14 drinks
Women	3 drinks	7 drinks
***Questions used to define drinking above US recommended limits***		
	> Weekly limits	AUDIT-C Questions #1-2
	Heavy episodic drinking	AUDIT-C Questions #2-3

^*^Drinks refer to standard-sized drinks: 12 oz. beer, 5 oz. wine, or 1.5 oz. liquor.

^**^The same definition of heavy episodic drinking was used in men and women because the AUDIT-C questions used in this study did not assess drinking 4 or more drinks on an occasion.

Several brief screens for identifying patients with unhealthy alcohol use are recommended based on validation studies [3,4]. These validation studies have identified optimal screening thresholds for unhealthy alcohol use by comparing alcohol screens to a gold standard measure of unhealthy alcohol use obtained from independent, detailed interview assessments of both alcohol consumption and symptoms of AUD [6,7]. The optimal screening thresholds maximize both sensitivity and specificity for unhealthy alcohol use based on the gold standard measure, irrespective of whether patients report risky drinking and/or symptoms of AUD on the brief alcohol screen.

The 3-item Alcohol Use Disorders Identification Test–Consumption (AUDIT-C) questionnaire is one of several brief screens for unhealthy alcohol use recommended by the USPSTF and consists of the first three questions of the World Health Organization’s 10-item AUDIT, which ask about alcohol consumption [8]. The AUDIT-C, scored 0–12 points, is a scaled marker of alcohol consumption, as well as the risk of AUD and other complications of drinking [9-21]. The AUDIT-C has been extensively validated in a wide variety of settings and populations, including primary care patients and general population samples in the US, Belgium, Spain, Germany, Brazil, and Taiwan [6,7,22-32], and is increasingly being integrated into routine preventive care [3,33-35]. The optimal AUDIT-C thresholds for unhealthy alcohol use in the US, based on comparison to detailed gold standard interviews, are ≥4 points for men and ≥3 points for women [6,7,22,36].

However, a potential limitation of the AUDIT-C is that there can be inconsistencies between the AUDIT-C screen result—positive or negative based on the total AUDIT-C score (≥4 and ≥3 points for men and women, respectively)—and whether respondents report drinking above or below US recommended drinking limits on the AUDIT-C questions [33]. These inconsistencies can happen in two general ways: patients can screen positive for unhealthy alcohol use based on the AUDIT-C score while reporting drinking below US recommended limits or they can screen negative based on the AUDIT-C score while reporting drinking above recommended drinking limits. As above, the optimal thresholds for a positive AUDIT-C score were determined by validation studies based on detailed, independent interviews about alcohol consumption and AUD symptoms, irrespective of these inconsistencies. Moreover, these inconsistencies are not necessarily “false positive” or “false negative” screens because the individual AUDIT-C questions underestimate typical drinking [37] and do not assess AUD symptoms. Nevertheless, these inconsistencies can be *perceived* by clinicians to be false positive or false negative screens and may result in uncertainty about whether or not to offer a BI for unhealthy alcohol use. However, no prior study to our knowledge has assessed whether these inconsistencies are common, or how often they occur in practice. If they were common, clinicians would need to be prepared to interpret them and provide further assessment of alcohol use and/or offer BIs appropriately.

This study described how often the results of screening with the AUDIT-C—positive or negative for unhealthy alcohol use *based on the AUDIT-C score*—are inconsistent with whether the individual’s *reported drinking* on the AUDIT-C is above or below US recommended drinking limits (Table 1). We determine the prevalence of these inconsistencies in two national US samples of individuals who reported drinking alcohol in the past year.

## Methods

### Overview

This study used two national US surveys that included the AUDIT-C to describe how often the results of screening—*positive or negative screen based on the total AUDIT-C score*—were inconsistent with the *reported drinking*—above or below US recommended drinking limits—on the same AUDIT-C. This study is therefore different from validation studies of the AUDIT-C, which compare the AUDIT-C to independent, gold-standard measures of unhealthy alcohol use. Instead, the present study was designed to assess how often inconsistencies between the results of the screen and reported drinking on the AUDIT-C questions occur in large samples of people who drink alcohol.

### Data sources and study population

#### US general population sample

This study used secondary data from the 2001–2002 National Epidemiologic Survey on Alcohol and Related Conditions (NESARC), which evaluated the prevalence of alcohol use disorders and related disabilities in a general US population sample of 43,093 civilian, non-institutionalized adults 18 years of age and older using computer-assisted personal interviews (81% response rate) [38]. Young adults (18–24), non-Hispanic blacks, and Hispanics were oversampled to facilitate subgroup analyses [38]. NESARC participants were asked if they had had at least one drink of alcohol in their entire lives and, if so, if they had at least one drink in the past 12 months. If participants answered no to either question, they were considered non-drinkers by NESARC and were not asked about past-year alcohol use. Non-drinkers represented 34.6% of the NESARC sample, with 17.3% lifetime abstainers and 17.3% former drinkers [39]. Respondents who reported past-year drinking were asked about their alcohol use in the past year using a version of the AUDIT-C that has been previously validated (Table 2) [23,24].

**Table 2:** AUDIT-C versions used in this study

	
**US General Population Sample (2001–2002 NESARC Surveys) **[24]	
AUDIT-C Question #1	**During the last 12 months, about how often did you drink ANY alcoholic beverage?** Never (0); 1 or 2 times in the last year (1); 3 to 6 times in the last year (1); 7 to 11 times in the last year (1); Once a month (1); 2 to 3 times a month (2); Once a week (2); 2 times a week (3); 3 to 4 times a week (3); Nearly every day (4); Every day (4)
AUDIT-C Question #2	Counting all types of alcohol combined, how many drinks did you USUALLY have on days when you drank during the last 12 months?* 1 or 2 (0); 3 or 4 (1); 5 or 6 (2); 7 to 9 (3); 10 or more (4)
AUDIT-C Question #3	**During the last 12 months, about how often did you drink FIVE OR MORE drinks in a single day?** Never (0); 1 or 2 times in the last year (1); 3 to 6 times in the last year (1); 7 to 11 times in the last year (1); Once a month (2); 2 to 3 times a month (2); Once a week (3); 2 times a week (3); 3 to 4 times a week (3); Nearly every day (4); Every day (4)
**VA Outpatient Sample (2004–2007 SHEP Surveys)**	
AUDIT-C Question #1	**How often have you had a drink containing alcohol *in the past 12 months*? Consider a “drink” to be a can or bottle of beer, a glass of wine, a wine cooler, or one cocktail or a shot of hard liquor (like scotch, gin, vodka).** Never (0); Monthly or less (1); 2 to 4 times a month (2); 2 to 3 times a week (3); 4 to 5 times a week (4); 6 or more times a week (4)
AUDIT-C Question #2	**How many drinks containing alcohol did you have on a *typical *** **day when you were drinking *in the past 12 months*?** 0 drinks (Did not drink in the past 12 months) (0 points); 1 to 2 drinks (0); 3 to 4 drinks (1); 5 to 6 drinks (2); 7 to 9 drinks (3); 10 or more drinks (4).
AUDIT-C Question #3	**How often did you have *6 or more *** **drinks on one occasion *in the past 12 months*?** Never (0 points); Less than monthly (1); Monthly (2); Weekly (3); Daily or almost daily (4).

*Question #2 in the NESARC AUDIT-C was based on an open-ended response and then mapped onto these AUDIT-C response options.

NESARC participants who were past-year drinkers and responded to all three questions of the AUDIT-C (99.4% of drinkers) were eligible for this study and are referred to as the “US general population sample” hereafter.

#### VA outpatient sample

This study also used secondary data from the VA’s national outpatient satisfaction survey (2004–2007), called the Survey of Healthcare Experiences of Patients (SHEP). SHEP is conducted by the VA Office of Analytics and Business Intelligence and is mailed monthly to a random sample of outpatients who have had a past-month outpatient VA visit and who have not completed SHEP in the prior 12 months. Of those who were mailed SHEP during the study period, 1,016,774 responded, and a 70% response rate has been reported previously over a similar time frame [40].

SHEP respondents were eligible for the present study if they completed all three AUDIT-C questions (87.3% of responders) and reported drinking alcohol in the past year (52.7% of AUDIT-C completers) [6,33]. This sample is referred to as the “VA outpatient sample.” Past-year non-drinkers were excluded so that the sample would be comparable to the US general population sample.

### Measures

#### The AUDIT-C

Each question of the AUDIT-C is scored 0 to 4 points, and questions are summed for a total AUDIT-C score of 0–12 points, with 0 indicating no alcohol use in the last year and 12 indicating the most severe unhealthy alcohol use. Several different versions of the AUDIT-C have been evaluated in the US and the same cut-points for a positive screen for unhealthy alcohol use (≥4 points for men and ≥3 points for women) have been validated in clinical validation studies irrespective of whether the AUDIT-C specified a past-year time frame or standard US drink sizes, or whether standard wording and/or standard response options for questions 1–3 of the original AUDIT were used [6,7,22,36]. The cut-point for women was also optimal irrespective of whether the third AUDIT-C question asked about the frequency of drinking 4 or more or 6 or more drinks on an occasion for women [22].

The two versions of the AUDIT-C used in this study differed slightly (Table 2), but both versions have been validated for identifying individuals with past-year unhealthy alcohol use using independent, in-depth interviews about alcohol consumption and AUD to define the gold standard of unhealthy alcohol use [6,7,22,24]. On both AUDIT-C versions, each AUDIT-C question is scored from 0–4, and the scores are summed for a total score of 0–12 (Additional file 1: Appendix A).

#### Screen results based on the total AUDIT-C score

Respondents were considered to screen positive for unhealthy alcohol use (on either the NESARC or SHEP versions of the AUDIT-C) if their total AUDIT-C score was ≥4 points for men or ≥3 points for women and negative if their total AUDIT-C score was 1–3 points for men and 1–2 points for women. These were the optimal thresholds for unhealthy alcohol use in VA outpatient samples [7,22] and non-VA primary care patients [6] and among NESARC participants who reported drinking [23,24].

#### Reported drinking above US recommended limits on the AUDIT-C questions

Individuals were considered to report drinking above US recommended limits on the AUDIT-C if they reported (1) heavy episodic drinking defined as drinking above recommended drinking limits for any single day (≥5 drinks in a day) on AUDIT-C Questions #2 or #3 or (2) drinking above average weekly limits (>14 drinks a week for men or >7 drinks a week for women) based on AUDIT-C Questions #1 and #2. Although NIAAA recommendations include gender-specific limits for heavy episodic drinking (Table 1), this study used a single definition of heavy episodic drinking (≥5 drinks in a day) for both men and women because the SHEP survey AUDIT-C did not include gender-specific questions about heavy episodic drinking (i.e. AUDIT-C Question #3). Respondents’ average weekly consumption was calculated using the lower end of response ranges for reported number of drinking days per week (Question #1) and the number of drinks per typical drinking day (Question #2) to conservatively estimate the lowest possible reported weekly consumption. For example, an individual reporting drinking 2 to 4 times per month and 5 to 6 drinks per drinking occasion would be estimated to drink 5 drinks twice a month, or 2.5 drinks per week on average. A conservative interpretation of response ranges was used because instances where patients who screen positive on the AUDIT-C but who report drinking below recommended drinking limits have been most bothersome to clinicians [33]. Therefore, when estimating individuals’ consumption based on the individual AUDIT-C questions, we were interested in identifying *all possible* instances of reported drinking within recommended drinking limits and having a positive AUDIT-C screen.

#### Other measures

Age, gender, education, race/ethnicity, and marital status were available for both the US general population and VA outpatient samples.

### Analyses

***Inconsistent response patterns*** were defined as AUDIT-C response patterns yielding screening results based on the AUDIT-C score (positive or negative) that were inconsistent with reported drinking (below or above US recommended limits). All analyses were stratified by gender: male drinkers were evaluated separately from female drinkers. Inconsistent response patterns included those in which: a) the AUDIT-C screen was positive but the responses to the AUDIT-C questions indicated drinking within US recommended limits; and b) the AUDIT-C screen was negative but the responses to the AUDIT-C questions indicated drinking above US recommended limits. Descriptive statistics were used to evaluate the proportion of male and female drinkers in each sample (US general population sample and VA outpatient sample) who had inconsistent response patterns.

All analyses were conducted in Stata 11.2 [41]. This study received approval and waivers of written informed consent and HIPAA authorization from the VA Puget Sound Health Care System Institutional Review Board.

## Results

The US general population sample included 26,610 drinkers and the VA outpatient sample included 467,416 drinkers who met eligibility criteria for this study. Table 3 shows available demographic characteristics of the study samples. The VA sample was generally older and predominantly male. Despite age and gender differences, the prevalence of positive screens in these two samples of individuals who reported drinking in the past year were similar: 45% and 46% of male drinkers and 35% and 37% of female drinkers screened positive for unhealthy alcohol use at the optimal cut-points (≥4 points for men and ≥3 points for women) in the US general population and VA outpatient samples, respectively. Ten response patterns were identified that yielded inconsistent results between screening based on the total AUDIT-C score (positive or negative) and reported drinking (above or below US recommended limits) on the AUDIT-C questions (Table 4).

**Table 3:** Demographics of the US general population and VA outpatient samples

	**US general population sample**		**VA outpatient sample**	
	**(N = 26,610)**		**(N = 467,416)**	
**Demographic characteristic**	**n**	**(%)**	**n**	**(%)**
**Gender**				
Male	12,856	(48.3)	450,681	(96.4)
Female	13,754	(51.7)	16,735	(3.6)
**Age**				
18-29	6,073	(22.8)	4,477	(1.0)
30-39	6,325	(23.8)	8,619	(1.8)
40-49	5,699	(21.4)	25,994	(5.6)
50-59	3,923	(14.7)	102,506	(21.9)
60-69	2,331	(8.8)	119,871	(25.6)
70-79	1,557	(5.9)	137,491	(29.4)
80+	702	(2.6)	68,458	(14.6)
Education*				
High school/GED or less	10,582	(39.8)	211,501	(45.3)
Some college/Associate’s degree	8,619	(32.4)	146,837	(31.4)
College graduate or more	7,409	(27.8)	104,703	(22.4)
Marital status*				
Married/Living with significant other	14,122	(53.1)	305,107	(65.3)
Widowed	1,531	(5.8)	39,939	(8.5)
Divorced/separated	4,375	(16.4)	90,794	(19.4)
Never married	6,582	(24.7)	25,482	(5.5)
Race/ethnicity*				
American Indian/Alaska Native	410	(1.5)	9,756	(2.1)
Asian/Native Hawaiian/Pacific Islander	656	(2.5)	3,723	(0.8)
Black/African American	4,126	(15.5)	26,752	(5.7)
White	16,542	(62.2)	401,149	(85.8)
Hispanic/Latino	4,876	(18.3)	20,819	(4.5)
**Positive AUDIT-C screens (among drinkers)**				
Male (≥4 points)	5,903	(45.9)	202,003	(44.8)
Female (≥3 points)	4,783	(34.8)	6,202	(37.1)

*Column totals of these demographic characteristics for VA Outpatient Sample do not total to 100% due to missing values.

**Table 4:** Prevalence of inconsistency between screening results based on AUDIT-C scores and reported drinking on the same AUDIT-C in men and women

				**US general population sample**		**VA outpatient sample**	
				**(N = 26,610)**		**(N = 467,416)**	
**Men**							
**Positive screen based on AUDIT-C score**							
**AUDIT-C score**				**% reporting < recommended limits**			
**Total**	**Q#1**	**Q#2**	**Q#3**	**%**	**(95% CI)**	**%**	**(95% CI)**
**4**	3	1	0	2.3	(2.1–2.6)	1.0	(1.0-1.0)
**4**	4	0	0	5.4	(5.1–5.8)	12.9	(12.7–13.0)
**5**	4	1	0	1.1	(0.9–1.3)	1.9	(1.8–1.9)
**All inconsistent positive screens**				8.9*	(8.4–9.4)	15.8	(15.6–15.8)
**Negative screen based on AUDIT-C score**							
**AUDIT-C score**				**% reporting > recommended limits**			
**Total**	**Q#1**	**Q#2**	**Q#3**	**%**	**(95% CI)**	**%**	**(95% CI)**
**2**	1	0	1	1.1	(0.9–1.2)	2.0	(2.0–2.0)
**3**	1	1	1	2.0	(1.7–2.2)	0.6	(0.6–0.6)
**3**	1	0	2	0	--	0.3	(0.3–0.3)
**3**	1	2	0	0	--	0.1	(0.01-0.1)
**3**	2	0	1	1.9	(1.7–2.2)	2.3	(2.2–2.3)
**All inconsistent negative screens**				4.9	(4.6–5.3)	5.3	(5.2–5.3)
**All inconsistent negative & positive screens**				**13.8**	**(13.2–14.4)**	**21.1**	**(20.9–21.1)**
**Women**							
**Positive screen based on AUDIT-C score**							
**AUDIT-C score**				% reporting < recommended limits **			
**Total**	**Q#1**	**Q#2**	**Q#3**	**%**	**(95% CI)**	**%**	**(95% CI)**
**3**	2	1	0	2.6	(2.4–2.9)	1.5	(1.3–1.7)
**3**	3	0	0	8.2	(7.7–8.6)	8.1	(7.7–8.5)
**4**	3	1	0	1.6	(1.4–1.8)	0.6	(0.5–0.8)
**4**	4	0	0	5.0	(4.6–5.4)	7.2	(6.8–7.6)
**All inconsistent positive screens**				17.5*	(16.8–18.1)	17.4	(16.9–18.0)
**Negative screen based on AUDIT-C score**							
**AUDIT-C score**				**% reporting > recommended limits**			
**Total**	**Q#1**	**Q#2**	**Q#3**	**%**	**(95% CI)**	**%**	**(95% CI)**
**2**	1	0	1	0.8	(0.7–1.0)	3.3	(3.0–3.6)
**All inconsistent negative screens**				0.8	(0.7–1.0)	3.3	(3.0–3.6)
**All inconsistent negative & positive screens**				18.3*	**(17.7–18.9)**	**20.7**	**(20.1–21.3)**

*Numbers do not add up to totals due to rounding.

**All negative screens with report of risky drinking were due to heavy episodic drinking (≥5 drinks in a day).

### Prevalence of inconsistent response patterns

Overall, among men in the US general population who reported drinking in the last year, 13.8% (95% CI 13.2–14.4) had inconsistent response patterns (Table 4). For men in the VA outpatient sample who reported drinking in the last year, 21.1% (CI 20.9–21.1) had inconsistent response patterns. Among women, 18.3% (CI 17.7–18.9) and 20.7% (CI 20.1–21.3) had inconsistent response patterns in the US general population and VA outpatient samples, respectively (Table 4).

### Inconsistent response patterns in men

#### Positive AUDIT-C screens and report of drinking below US recommended limits

Among male drinkers, positive AUDIT-C screens despite reporting drinking below recommended limits were the most common inconsistent response patterns, with 8.9% (CI 8.4–9.4) in the US general population sample and 15.8% (CI 15.6–15.8) in the VA outpatient sample (Table 4). The most common inconsistent response pattern among men in both the US general population and VA outpatient samples was report of drinking 4 or more days a week (4 points), 1 to 2 drinks per drinking day (0 points), and never engaging in heavy episodic drinking (0 points)—or a 4-0-0 pattern (Table 4).

#### Negative AUDIT-C screens and report of drinking above US recommended limits

Among male drinkers in the US general population sample, 4.9% (CI 4.6–5.3) had negative screens despite reporting heavy episodic drinking (Table 4). Among male drinkers in the VA sample, 5.3% (CI 5.2–5.3) had negative AUDIT-C screens despite reporting heavy episodic drinking (Table 4).

### Inconsistent response patterns in women

#### Positive AUDIT-C screens and report of drinking below US recommended limits

Among female drinkers, 17.5% (CI 16.8–18.1) of the US general population sample and 17.4% (CI 16.9–18.0) of the VA outpatient sample had positive AUDIT-C screens based on the total score despite reported drinking below recommended limits on the AUDIT-C questions (Table 4). The most common inconsistent response pattern among women in both the US general population and VA outpatient samples was drinking 2 to 3 times a week (3 points), 1 to 2 drinks per drinking day (0 points), and never engaging in heavy episodic drinking (0 points), or a 3-0-0 pattern (Table 4).

#### Negative AUDIT-C screens and report of drinking above US recommended limits

Among female drinkers, 0.8% (CI 0.7–1.0) of the US general population sample and 3.3% (CI 3.0–3.6) of the VA sample had negative AUDIT-C screens despite reporting heavy episodic drinking (Table 4).

## Discussion

Inconsistencies between results of alcohol screening—positive or negative based on the total AUDIT-C score—and reported drinking above or below US recommended limits on the same AUDIT-C screen can confuse clinicians [33]. However, the prevalence of these inconsistencies has not been previously described. In this study of individuals who reported drinking alcohol in the past year, 14% of men in the US general population sample and 21% of men in the VA outpatient sample, as well as 18% of women in the US general population sample and 21% of women in the VA outpatient sample had screening results based on the AUDIT-C score that were inconsistent with reported drinking on the same AUDIT-C. It is important for clinicians to be aware of these inconsistencies when offering screening and BI for unhealthy alcohol use.

The majority of inconsistent response patterns in both samples for men and women were positive screens based on the total AUDIT-C score despite report of drinking below US recommended limits on the same AUDIT-C. These results should not be assumed to be false positive screens, as patients may have under-reported their alcohol consumption or have an AUD (a diagnosis that does not require surpassing a drinking threshold). In a previous VA study, 46% of men who drank over 14 drinks a week based on an independent, gold standard interview reported drinking below 14 drinks a week on AUDIT-C questions #1-2 [37]. Women may under-estimate their alcohol consumption even more often than men due to stigma associated with unhealthy alcohol use [42]. While patients may be accurately reporting their perception of their drinking on the AUDIT-C questions, they often under-estimate their true consumption compared to in-depth assessments that ask about different beverage types separately and take into account typical drink size and alcohol content [43]. For example, female VA patients report alcohol-related problems—tolerance, blackouts, and feeling the need to cut down—at total AUDIT-C scores as low as 3 [44], which are associated with report of drinking within US recommended limits (Table 4). If providers erroneously consider positive AUDIT-C screens as “false positives” and do not to provide BIs to such patients, some who would benefit from BI will be denied those benefits.

Although the majority of inconsistent screening patterns were positive AUDIT-C screens despite report of drinking below US recommended limits, up to 5% of men and 3% of women had negative AUDIT-C screens despite reported heavy episodic drinking (ie. drinking above recommended daily limits). This emphasizes the value of reviewing both the individual responses, particularly AUDIT-C question #3 (frequency of heavy episodic drinking), as well as the total AUDIT-C score. Patients with heavy episodic drinking despite negative screens should ideally be offered BIs and advised about risks associated with heavy episodic drinking.

Even though providers using the AUDIT-C may face the above inconsistent response patterns, the AUDIT-C has certain advantages. The AUDIT-C has been validated as a screen for the spectrum of unhealthy alcohol use in diverse patient populations [6,7,22-32]. It also assesses whether patients drink at all (AUDIT-C scores > 0) and identifies and documents their typical drinking patterns, an advantage over single-item screens [3]. In addition, the AUDIT-C score is a scaled marker of risk [9-21]. As a result, the AUDIT-C is commonly used [33-35].

Providers who work in healthcare settings that use the AUDIT-C therefore need to know how to adapt their BIs with patients who may have an inconsistent response pattern. Ideally, all patients who screen positive for unhealthy alcohol use should undergo further assessment regarding patterns of alcohol consumption and AUD symptoms [39]. One way of assessing alcohol-related symptoms would be to ask patients the other seven AUDIT questions [8]—the AUDIT-C represents the first three questions—to identify alcohol-related consequences patients may report having experienced in the past year. Alternatively, because some systems may not have resources to conduct detailed assessments of all screen-positive patients, it might be appropriate to offer these patients BIs and reserve assessment for patients with higher AUDIT-C scores or those who do not respond to a BI [35]. For such individuals who report drinking below recommended limits despite screening positive, providers can recommend that patients *maintain* their drinking below recommended limits, specifically informing patients of these limits and standard drink sizes, and educate patients about the health consequences of exceeding these limits [3].

Patients with heavy episodic drinking despite negative screening results based on the AUDIT-C score can be identified by computerized algorithms in electronic health records or clinician review of responses to the AUDIT-C. These patients can be complimented on the fact that they drink below *weekly* limits, and provided with feedback on the risks of heavy episodic drinking, which is strongly associated with development of alcohol dependence [45,46] and a variety of other health concerns [12,47-57]. Finally, such patients can be offered explicit advice to limit the number of drinks they have in any single day.

### Limitations

This study used two samples of drinkers, a US general population sample and a VA outpatient sample, but generalizability to other samples of drinkers is unknown. This study did not include an independent interview gold standard for unhealthy alcohol use and therefore cannot address how often observed inconsistencies represented false positive or negative screens. This study’s conservative approach to defining reported drinking based on the lower end of response ranges could have underestimated reported drinking levels thereby potentially exaggerating inconsistencies between positive scores and reported drinking within limits. In addition, the AUDIT-C question about heavy episodic drinking may miss more women than men because the definition of reported drinking above recommended daily limits that was used in this study (≥5 drinks in a day) is higher than the US gender-specific recommended daily limit for women (≥4 drinks per day). Further, Question #3 of the AUDIT-C used in the US general population sample asked how often the respondent drank 5 or more drinks in a single day, as is considered heavy episodic drinking in the US [8,39], whereas the AUDIT-C in the VA outpatient sample (Table 2) asked about the frequency of 6 or more drinks on an occasion, as in the original validated AUDIT-C [7]. Differences in the sample selection for the two surveys as well as demographic characteristics could account for discrepancies observed in the exact prevalence of particular inconsistent response patterns. Nevertheless, these findings make it clear that screening positive based on AUDIT-C total score despite report of drinking below US recommended limits on AUDIT-C questions is not an uncommon occurrence in clinical or general population samples of drinkers.

## Conclusion

The AUDIT-C is a widely used screen for unhealthy alcohol use that has been validated in various patient populations. However, individuals can screen positive based on the total AUDIT-C score despite report of drinking below US recommended limits on the AUDIT-C questions, and they can screen negative despite reporting heavy episodic drinking. This report of the prevalence of these inconsistencies—which is the first such report to our knowledge—demonstrates that some patterns are fairly common among individuals who drink alcohol. These results suggest that both the score and reported alcohol consumption should be taken into account when offering BIs after AUDIT-C screening.

## Competing interests

The authors declare that they have no competing interests.

## Authors’ contributions

Author KAB conceived of the study, obtained funding, and oversaw all aspects of the study. Authors ADR, GTL and LC undertook the statistical analyses. Authors KED and AL managed the literature searches and wrote the first draft of the paper. All authors have contributed to and have approved the final manuscript.

## Supplementary Material

Additional file 1: Appendix A Sensitivity and specificity of AUDIT-C for identifying unhealthy alcohol use (clinical samples) or drinking above US recommended limits (NESARC). Click here for file
