# Supplementary material for: Inconsistencies between alcohol screening results based on AUDIT-C scores and reported drinking on the AUDIT-C questions: prevalence in two US national samples
Source: Addict Sci Clin Pract. 2014 Jan 27;9(1):2. doi: 10.1186/1940-0640-9-2 (PMC3946205; doi:10.1186/1940-0640-9-2)
Supplement: Additional file 1: Appendix A — Sensitivity and specificity of AUDIT-C for identifying unhealthy alcohol use (clinical samples) or drinking above US recommended limits (NESARC). [file 1940-0640-9-2-S1.pdf]

Appendix A. Sensitivity and Specificity of AUDIT-C for Identifying Unhealthy Alcohol Use (Clinical Samples) or Drinking Above U.S. Recommended Limits (NESARC)

	VA Outpatient Population* (standard AUDIT-C)		Non-VA Outpatient Population† (standard AUDIT-C)		U.S. General Population‡ (NESARC AUDIT-C)	
Women	Sensitivity %	Specificity %	Sensitivity %	Specificity %	Sensitivity %	Specificity %
≥2	81	96	89	78	--	--
≥3	60	96	73	91	96	80
≥4	38	98	57	96	81	93
Men						
≥3	95	60	92	79	100	58
≥4	86	72	86	89	99	79
≥5	68	90	72	96	91	95

*Bradley, 2003 Criterion standard was unhealthy alcohol use (using DSM-IV criteria for AUD); Bush, 1998 (using DSM-III-R criteria for AUD)

†Bradley, 2007 Criterion standard was unhealthy alcohol use (DSM-IV criteria for AUD)

‡Dawson, 2005b Criterion standard was drinking above U.S. Recommended Limits
